# Fracture of the Tibia, with Avulsion of the Internal Malleolus, and Penetrating Wound of the Ankle-joint; Consecutive Abscesses; Necrosis of the Tibia, and Resection of Its Inferior Extremity—Recovery

**Published:** 1842-07

**Authors:** 


*Fracture of the tibia, with avulsion of the internal malleolus, and penetrating wound of the ankle-joint; consecutive abscesses; necrosis of the tibia, and resection of its inferior extremity—recovery.* By M. Castella.—C. H. J. St. Croix, aged 42, was thrown down on the 22d of May, 1840, under a baggage wagon. The right foot was turned forcibly outwards, the internal malleolus was torn from its attachment, and the inferior extremity of the tibia protruded through the skin to a considerable extent.

Considerable inflammation followed the injury, abscesses formed behind the tibia, and amputation being deemed necessary, the patient was conveyed to the hospital at Pourtales, on the 27th of May.

The right foot was found to be very much averted—the fibula was fractured three inches above the external malleolus, which was flattened and crepitated on pressure—and above which the fibula presented a reentrant obtuse angle. Internally the inferior extremity of the tibia, black and denuded, projected to the extent of two inches, and the internal malleolus was torn off at the level of the articular surface of the tibia. Several fistulous openings existed at the posterior border of the tibia, and yielded a fetid sanious discharge. The foot was œdematous; there was considerable fever, and the patient was very much debilitated.

June 2d.—Before resorting to amputation, it was determined to resect a portion of the necrosed tibia, to replace the foot in its natural position, and to retain it in situ by means of Dupuytren's apparatus for fracture of the fibula.

With this view, an incision was made anteriorly and posteriorly to the tibia, and a slip of lead passed round the external surface of the bone, as high up as possible, to protect the soft parts, and especially the vessels; the bone was then sawed across, and twenty-one lines of its inferior extremity removed.

The foot was now easily drawn inwards, and kept in position, with Dupuytren's bandage; the depression of the fibula disappeared; and the leg being semi-flexed, was placed on its outer surface.

Phlegmonous inflammation followed the operation, requiring several incisions which gave issue to sloughy cellular tissue; but at length granulations covered the inferior extremity of the tibia; the medullary membrane became rapidly developed, and in proportion as the vacant space between the tibia, fibula, and astragalus became filled with granulations, the heel which had been drawn forcibly upwards by the action of the gastrocnemii and solei muscles, resumed its situation, and the foot ceased to be extended on the leg.

At the expiration of two months the fibula was consolidated, and presented a symmetrical outline; and after a few months, the wound was completely consolidated. The foot had acquired some small degree of motion, and the space resulting from the resection of the tibia had disappeared, and was filled by a solid bony substance. In October the patient began to walk on crutches; and on the 11th October he left the hospital, being able to walk with the aid of a stick.—*Dublin Med. Press*, from *Gazette Médicale de Paris.*

[This case resembles in some respects that of Wm. Smith, which was noticed in the February number of our Journal, p. 141. The mode of treatment adopted is likewise similar to that so successfully pursued in the latter by Dr. Shipman, of Cortlandville, N. Y. We may mention, by the way, that a correspondent writes us that Smith is entirely well, and has walked 30 miles in one day since last winter; his injured limb, save that it is shorter, being equally as serviceable as the other. Dr. Shipman's triumph over his persecutors, or rather we should say, the triumph of science and skill over ignorance and dulness, is therefore complete. Thus may it ever be. We commend the case above quoted to the especial notice of Professors Hamilton and Webster of the Geneva Medical College, and to all others who can do nothing without "authority from books."—Eds.]

